# Accuracy and Reliability of AI Models in Emergency Myocardial Infarction Education

**DOI:** 10.1155/emmi/5530861

**Published:** 2026-06-13

**Authors:** İbrahim Korkmaz

**Affiliations:** ^1^ Emergency Department, Izmir City Hospital, Izmir, Turkey

**Keywords:** artificial intelligence, education of patients, emergency service, hospital, myocardial infarction

## Abstract

**Background:**

Acute myocardial infarction (AMI) is a major global cause of morbidity and mortality. Large language models (LLMs) are emerging tools for patient education. This study evaluated the performance of three LLMs in delivering accurate, reliable, and readable educational content regarding AMI.

**Methods:**

In this cross‐sectional study (February–March 2025), a clinical case of a patient with an inferior STEMI ECG was presented to three LLMs: ChatGPT‐4o, Claude 3.7 Sonnet, and Gemini Advanced 2.0 Flash. Each model answered 30 patient‐focused questions across three domains: general disease knowledge, diagnostic processes, and treatment approaches. Responses were assessed by four emergency medicine associate professors (10–20 years of experience) using a 5‐point Likert scale for accuracy, DISCERN and EQIP tools for reliability and quality, and standard readability indices.

**Results:**

ChatGPT‐4o achieved the highest accuracy score (4.38 ± 0.38), followed by Claude 3.7 (4.09 ± 0.55) and Gemini 2.0 (3.92 ± 0.41) (*p* < 0.001). ChatGPT‐4o performed significantly better in general information (*p* = 0.002) and diagnostics (*p* = 0.009), while Claude 3.7 excelled in treatment‐related content (*p* = 0.015). Claude 3.7 produced significantly more readable responses than both ChatGPT‐4o and Gemini 2.0 across all indices (Flesch–Kincaid, *p* = 0.002, Gunning Fog, *p* ≤ 0.001, Coleman–Liau, *p* = 0.003). ChatGPT‐4o scored “excellent” on the DISCERN scale; all models were rated as “good quality with minor shortcomings” on EQIP. Reliability scores did not differ significantly (DISCERN, *p* = 0.188; EQIP, *p* = 0.935).

**Conclusions:**

LLMs show promise in supporting patient education on AMI. While ChatGPT‐4o offers superior accuracy and reliability, Claude 3.7 enhances accessibility through clearer language. This is the first study comparing three LLMs for AMI education in an emergency context, underscoring that physician oversight remains essential for educational applications in emergency medicine.

## 1. Introduction

Acute myocardial infarction (AMI) remains a significant health concern globally due to its high rates of illness and death. The World Health Organization (WHO) reports that cardiovascular diseases were responsible for nearly 17.9 million deaths in 2019, and AMI is known to contribute substantially to this burden [1]. AMI is usually associated with a thrombotic event in the background of atherosclerotic coronary artery disease (CAD). High blood pressure, high cholesterol, diabetes, smoking, and having relatives with heart disease are among the main risk factors for AMI. The 2023 European Society of Cardiology (ESC) guidelines emphasize the importance of obtaining a 12‐lead electrocardiogram (ECG) within the first 10 min, utilizing high‐sensitivity cardiac troponin assays, and performing early risk stratification [2]. Employing a multidisciplinary approach and maintaining a door‐to‐balloon time under 90 min are critical for minimizing myocardial injury [3]. AMI is of great importance in the emergency department (ED) because the time from symptom onset determines the extent of myocardial damage. In ED, rapid evaluation and referral of patients with chest pain to appropriate centers are vital.

Large language models (LLMs) are rapidly evolving artificial intelligence (AI) technologies that have transformative potential in healthcare education. In patient education, they can simplify complex medical information and provide clear, individualized explanations that support informed decision‐making [4]. Prior studies have demonstrated the utility of LLMs in managing chronic diseases, improving treatment adherence, and enhancing health literacy [5]. Furthermore, the continuous availability of LLM tools can reduce the burden of healthcare professionals and provide patients with uninterrupted access to reliable educational resources [6]. As such, LLMs are increasingly recognized as valuable tools in the digital transformation of patient education. However, issues such as ethics, reliability, and accuracy need to be carefully considered when using these technologies in educational contexts.

To date, no study has compared the three latest LLMs—ChatGPT‐4o, Claude 3.7 Sonnet, and Gemini Advanced 2.0 Flash—in the context of AMI patient education in the ED. In this study, a clinical scenario involving a patient with an inferior ST‐elevation myocardial infarction (STEMI) was presented to these state‐of‐the‐art LLMs. Frequently asked questions by patients and their relatives in the emergency setting were submitted to each model for evaluation. The responses were assessed for accuracy, reliability, and readability to determine which model would be the most suitable and practical for patient education purposes.

## 2. Material and Method

### 2.1. Study Design and Scope

This study evaluated the educational accuracy and consistency of AI language models in generating responses to emergency myocardial infarction scenarios. It is important to emphasize that our evaluation focused on the models’ performance as educational tools and knowledge repositories, not as clinical decision‐making agents. The assessment criteria were designed to measure content accuracy, guideline adherence, and pedagogical value rather than clinical deployment readiness.

### 2.2. Questions and Answers

This cross‐sectional study was conducted between February and March 2025. In the study, an example scenario of a patient diagnosed with AMI and related electrocardiography (ECG) data was uploaded to three different LLMs for evaluation. In the scenario, the patient addresses the LLM as follows: “Hello, I am the son of a 56‐year‐old male patient. My father has a history of diabetes and hypertension. He was brought to the emergency room about 2 h ago with severe chest pressure, pain radiating to his arm and jaw, nausea, and vomiting. The doctors have performed some blood tests and taken an electrocardiogram, and they mentioned that a complete blood count, biochemistry, troponin, and CK‐MB tests are being done. He was given 300 mg of aspirin and 5000 units of heparin intravenously. We were also informed that an emergency coronary angiography is planned. I am very worried and have some questions. Could you please explain to me, at the level of an expert doctor, what is happening and what we should expect?”

This clinical scenario and the Inferior STEMI ECG image from the Life in the Fastlane website [7] were uploaded to three different LLMs (ChatGPT‐4o, Claude 3.7 Sonnet, and Gemini Advanced 2.0 Flash). Although ECG images were uploaded to each LLM interface, it remains unclear whether and how these models process visual data. Therefore, while the models were presented with both textual and visual input, their responses were evaluated based solely on the answers they provided, without assuming accurate image interpretation capabilities. Subsequently, 30 questions were developed within the context of this scenario, reflecting the most frequently encountered inquiries from patients and their relatives during the authors’ routine clinical practice in EDs (e.g., one of the questions was: “Is it normal to have a heart attack at his age?”). Questions were derived from patient education materials available on official websites (e.g., NHS, Mayo Clinic, Cleveland Clinic), patient forums, and structured interviews with clinicians regarding common patient concerns. These questions were organized under three main headings—general information about the disease, diagnostic process, and treatment process—with 10 questions in each category. Although no formal psychometric validation was conducted, the questions were reviewed by the four emergency medicine associate professors who also served as evaluators, providing a degree of content verification by domain experts. The absence of formal validation with patient and family end‐users is acknowledged as a limitation of this study. The questions targeted a range of cognitive demands, including factual recall (e.g., “What is a heart attack?”), application of knowledge (e.g., “Why was aspirin given?”), and synthesis of clinical information (e.g., “What should we expect during recovery?”). In addition to clinical experience, publicly available patient information materials from healthcare institution websites were also reviewed to ensure the relevance and comprehensiveness of the selected questions. The complete prompt template and representative unedited model responses are provided in the Supporting File (Sections 1–2), and the full question list with item‐level accuracy scores is presented in Supporting Table S1. The GAI tools evaluated in this study were used exclusively as research subjects. Compliance with the GAMER reporting recommendations is summarized in Supporting Table S2. They were not employed in any phase of study design, data analysis, or manuscript preparation.

The evaluation was conducted on February 25, 2025. All three models were accessed via their official front‐end web interfaces (ChatGPT‐4o: chat.openai.com; Claude 3.7 Sonnet: claude.ai; Gemini Advanced 2.0 Flash: gemini.google.com). Default model parameters, including temperature settings, were applied without modification. All LLM sessions were initiated fresh for each question to prevent conversation history from influencing subsequent responses. Pre‐prepared questions were initially presented to each LLM individually and later simultaneously. However, to ensure methodological consistency, only the first responses provided during the individual questioning were recorded and included in the analysis. Although simultaneous presentation was performed, it was not intended for formal comparison, and no statistical evaluation was conducted between the two methods. The responses were reviewed for spelling and grammatical errors prior to evaluation, and no significant errors affecting the integrity of meaning were detected. No word or character limits were applied to allow the models to provide unrestricted and comprehensive answers. No real patient data or personally identifiable information was entered into any LLM interface; the clinical scenario was entirely fictional and constructed by the author for research purposes, and the ECG image was sourced from a publicly available educational resource. This study was conducted in accordance with the principles of the Declaration of Helsinki and was approved (Date/Approval No: 2026/144). Informed consent was obtained from all evaluators who participated in the assessment of AI‐generated responses.

### 2.3. Evaluation Metrics

The educational accuracy of the responses was evaluated by comparing them with current acute coronary syndrome guidelines [2, 3]. This evaluation was conducted by a panel of four associate professors with 10–20 years of experience in emergency medicine, who are directly responsible for the initial management of AMI patients in their clinical practice, including performing life‐saving interventions, initiating diagnostic evaluations, and determining initial treatment strategies such as medication administration and decisions regarding emergency coronary angiography. The assessors were blinded to the identity of the LLMs providing the responses. Blinding was achieved by presenting all responses to the evaluators with model identifiers removed and replaced with anonymous codes (Model A, Model B, Model C). The coding key was held solely by the corresponding author and was not disclosed to the evaluators until all assessments were completed. A 5‐point Likert scale, commonly used in previous studies evaluating LLMs, was employed to assess accuracy [8]. Scoring was done as follows:

1. Strongly disagree: Very bad or unacceptably inaccurate, involving a risk of serious harm.
2. Disagree: Low accuracy, contains potentially harmful errors.
3. Neither agree nor disagree: Moderately inaccurate, may be misunderstood but low risk of harm.
4. Agree: Good‐quality responses, except for small, harmless inaccuracies.
5. Strongly agree: Very high accuracy, no inaccuracies, no risk of harm.

All assessors initially evaluated the responses independently without consulting each other. In cases of disagreement, they held group discussions to reach a consensus based on current clinical guidelines. Since the main objective of the study was to examine the accuracy, comprehensibility, and practical usability of the LLMs’ responses to a Turkish‐speaking patient, all questions were posed in Turkish, and the models’ responses were evaluated in Turkish. When possible translation errors or the presence of foreign‐language words were observed, their impact on the integrity of the sentence meaning was taken into account during scoring.

Two validated instruments—DISCERN and ensuring quality information for patients (EQIP)—were employed to evaluate the reliability and informational quality of the responses. DISCERN, originally introduced by Charnock et al. in 1999, is designed to assess the quality of written information related to healthcare [9]. This scale focuses specifically on the reliability of information and the relevance of content for treatment. It consists of 16 items in total, and each item is scored from 1 to 5, with a score of 1 indicating serious deficiencies and a score of 5 indicating high quality. The first eight questions assess the reliability of the information, the next seven assess the content of the information on treatment, and the last item asks about overall quality. Excluding the last question, the total score range is between 16 and 75. The resulting scores are divided into five categories according to the level of quality: Excellent (63–75), Good (51–62), Fair (39–50), Poor (27–38), and Very Poor (16–26).

EQIP is a comprehensive evaluation framework developed by healthcare professionals and experts to assess the content of patient information materials [10]. This tool consists of 20 items focusing on various dimensions such as accuracy, objectivity, structural integrity, and design. Each item is scored as “yes,” “partially,” “no,” or “not specified,” with a maximum total score of 100 points. According to the total score obtained, content with a score between 76% and 100% is considered “well written and of very high quality”; a score between 51% and 75% is considered “of good quality with minor deficiencies”; a score between 26% and 50% is considered “having serious problems in terms of quality”; and a score between 0% and 25% is considered “of very low quality” [10]. Unlike DISCERN, no fixed score categories are defined for EQIP; the assessment is based on overall quality.

The consistency among the four evaluators was assessed using the intraclass correlation coefficient (ICC). The average‐measure ICC(A,4) was 0.584 (95% CI 0.456–0.688; *p* < 0.001), indicating moderate agreement among the raters. This level of agreement is considered acceptable for exploratory studies involving complex clinical judgments, though it also reflects the inherent subjectivity in evaluating AI‐generated educational content. Future studies may benefit from more structured scoring rubrics or additional rater training to improve inter‐rater reliability.

In addition, three different readability metrics were used to determine the comprehensibility level of the responses. The Flesch–Kincaid readability formula provides an estimate of grade level by taking into account the average sentence length and word difficulty in the text [11]. The Gunning Fog Index measures whether the text is clear and simple and usually expresses the result with a score from 0 to 20 [12]. The Coleman–Liau index, on the other hand, tries to determine the reading level of the text with an approach based on word and letter counting [13]. Higher readability scores indicate that a higher level of education is needed to understand the text, while lower readability scores indicate that simpler, clearer, and less complex texts [11–13]. Responses for each major language model were analyzed collectively according to these criteria. DISCERN, EQIP, and readability scores were calculated separately for each model, thus providing a comprehensive assessment of the overall performance of each LLM. It should be noted that the standard readability indices were originally developed for English texts and may have limited validity when applied to Turkish‐language responses. This limitation was taken into account when interpreting the readability results.

### 2.4. Statistical Method

All statistical analyses were performed using the “stats” package of R software (Version 4.3.2, R Foundation for Statistical Computing, Vienna, Austria). The normality of the data distribution was assessed by the Shapiro–Wilk test. Descriptive statistics were presented as frequency (*n*) and percentage (%) for categorical variables and median [Q1–Q3] or mean ± standard deviation (SD) for numerical variables. The primary endpoint of this study was defined as the overall total accuracy score across all 30 questions assessed via a 5‐point Likert scale. Secondary endpoints included domain‐specific accuracy scores (general information, diagnostic process, treatment process), readability indices (Flesch–Kincaid, Gunning Fog, Coleman–Liau), and reliability scores (DISCERN, EQIP). Friedman’s test was used to compare the responses of the three LLM systems (ChatGPT‐4o, Claude 3.7, and Gemini 2.0) according to Likert‐scale scores, DISCERN and EQIP scores, and readability indices. In the analyses where significant differences were found, pairwise comparisons were made with the Wilcoxon signed‐rank test. The statistical significance level was accepted as *p* < 0.05.

Effect sizes were reported as Kendall’s W for the Friedman test and as *r* (*r* = *Z*/√N) for pairwise Wilcoxon comparisons, where *r* < 0.3 indicates a small, 0.3–0.5 a moderate, and > 0.5 a large effect. Hodges–Lehmann estimators with 95% confidence intervals were calculated for each pairwise comparison. Equivalence testing was not applied as the study demonstrated statistically significant differences between models. Detailed effect sizes and confidence intervals for all comparisons are provided in Supporting Tables S3–S5.

The sample size of 30 questions was determined based on three clinical domains of AMI patient education, with 10 questions allocated to each domain, consistent with similar exploratory studies in the literature. As this was an exploratory study, no a priori power calculation was performed.

## 3. Results

**FIGURE 1:**
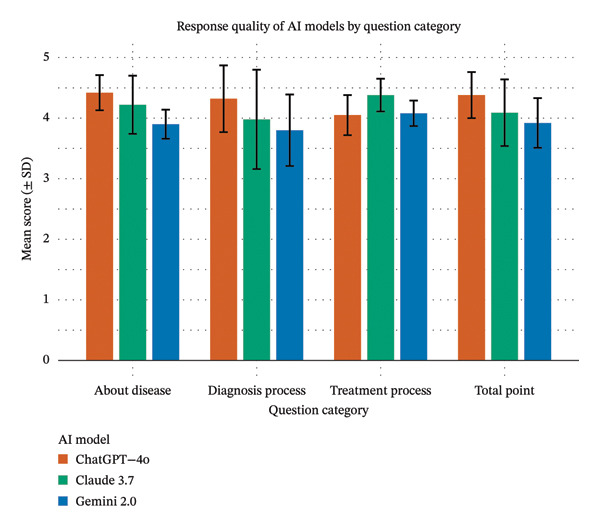
Average educational accuracy scores of the responses of three different large language models (ChatGPT‐4o, Claude 3.7 Sonnet, and Gemini Advanced 2.0 Flash) in the categories of information about the disease, diagnostic process, treatment process, and total score.

A total of 30 patient questions were posed to three different LLMs: ChatGPT‐4o, Claude 3.7, and Gemini 2.0, and the responses of each model were evaluated for educational accuracy, reliability, and readability. The mean total score was highest for the ChatGPT‐4o (4.38 ± 0.38), followed by the Claude 3.7 (4.09 ± 0.55) and Gemini 2.0 (3.92 ± 0.41) (Figure 1) (Table 1).

**TABLE 1:** Comparison of the educational accuracy scores of three different large language models (ChatGPT‐4o, Claude 3.7 Sonnet, and Gemini Advanced 2.0 Flash) in the categories of information about the disease, diagnostic process, treatment process, and total score.

Questions	ChatGPT‐4o	Claude 3.7 sonnet	Gemini advanced 2.0 flash	*χ* ^2^	*p* ^∗^	Effect size (W)
	Mean ± SD Median [Q1; Q3]	Mean ± SD Median [Q1; Q3]	Mean ± SD Median [Q1; Q3]			
General information about the disease	4.42 ± 0.29^a^	4.22 ± 0.48	3.90 ± 0.24	11.73	0.002^∗∗^	0.586
	4.38 [4.25; 4.50]	4.25 [4.06; 4.44]	4.00 [3.81; 4.00]			

Diagnosis process	4.32 ± 0.55^a^	3.98 ± 0.82	3.80 ± 0.59	9.29	0.009^∗∗^	0.465
	4.50 [4.25; 4.69]	4.25 [4.00; 4.44]	3.88 [3.50; 4.25]			

Treatment process	4.05 ± 0.33^a^	4.38 ± 0.27^b^	4.08 ± 0.21	8.4	0.015^∗∗^	0.420
	4.00 [3.81; 4.25]	4.50 [4.12; 4.50]	4.12 [4.00; 4.25]			

Total point	4.38 ± 0.38^a^	4.09 ± 0.55^b^	3.92 ± 0.41	26.81	< 0.001^∗∗^	0.447
	4.50 [4.25; 4.50]	4.25 [4.00; 4.25]	4.00 [3.75; 4.25]			

*Note:* a, b: refer to pairwise comparisons.

^a^ChatGPT‐4o vs Gemini 2.0.

^b^ChatGPT‐4o vs Claude 3.7.

^∗^Friedman test results.

^∗∗^significance at *p* < 0.05. Effect size is reported as Kendall’s W from the Friedman test.

When analyzed on the basis of headings, in the category of “General information about the disease,” the highest mean score was recorded in ChatGPT‐4o (4.42 ± 0.29) and the lowest score was recorded in Gemini 2.0 (3.90 ± 0.24). In the “diagnostic process” questions, the average score of ChatGPT‐4o was 4.32 ± 0.55, Claude 3.7 was 3.98 ± 0.82, and Gemini 2.0 was 3.80 ± 0.59. In the “treatment process” heading, the Claude 3.7 model had the highest mean of 4.38 ± 0.27, while ChatGPT‐4o had a mean of 4.05 ± 0.33, and Gemini 2.0 had a mean of 4.08 ± 0.21 (Figure 1, Table 1).

In the evaluation at the individual question level, the lowest scores of all three models were observed in the answers to the question “What does the ECG show? Is it normal?” (2.5 points for Gemini 2.0, 3.25 points for ChatGPT‐4o, and 1.75 points for Claude 3.7). The questions with the highest mean scores were: “Will a stent be inserted during angiography?” “Am I at risk of having bypass surgery?” “When will I be back to normal after treatment?” “Will these tests be painful?” and “How long will it take?” (Supporting Table S1).

In the subcategory of general information about the disease, a difference was observed among the models (Friedman *χ* ^2^ = 11.73), with a large effect size (Kendall’s *W* = 0.586). Pairwise comparisons indicated that ChatGPT‐4o outperformed Gemini 2.0 (median difference = 0.50, 95% CI: 0.375–0.75, *r* = 0.879), reflecting a very large effect. Differences between ChatGPT‐4o and Claude 3.7 (median difference = 0.25, 95% CI: 0–0.625, *r* = 0.416) and between Claude 3.7 and Gemini 2.0 (median difference = 0.375, 95% CI: −0.125–0.75, *r* = 0.554) were smaller and less consistent (Table 1, Table S3).

In the diagnostic process subcategory, differences between models were observed (*χ* ^2^ = 9.29), with a moderate effect size (*W* = 0.465). ChatGPT‐4o showed higher scores than Gemini 2.0 (median difference = 0.75, 95% CI: 0.625–0.75, *r* = 0.81), indicating a large effect, whereas other pairwise differences were more modest (Table 1, Table S3).

In the treatment process domain, a difference was also observed (*χ* ^2^ = 8.4), with a moderate effect size (*W* = 0.42). ChatGPT‐4o demonstrated higher scores than both Claude 3.7 (median difference = 0.375, 95% CI: 0.25–0.50, *r* = 0.796) and Gemini 2.0 (median difference = 0.50, 95% CI: 0.125–0.625, *r* = 0.694), indicating large effects, while the difference between Claude 3.7 and Gemini 2.0 was negligible (median difference = 0, 95% CI: −0.25–0.25, *r* = 0.059) (Table 1, Table S3).

For overall total scores, a clear difference was observed among models (*χ* ^2^ = 26.82), with a moderate‐to‐large effect size (*W* = 0.447). ChatGPT‐4o outperformed both Claude 3.7 (median difference = 0.375, 95% CI: 0.25–0.50, *r* = 0.614) and Gemini 2.0 (median difference = 0.50, 95% CI: 0.50–0.625, *r* = 0.803), both representing large effects, whereas the difference between Claude 3.7 and Gemini 2.0 was smaller (median difference = 0.25, 95% CI: 0–0.50, *r* = 0.358) (Table 1, Table S3).

Readability analyses demonstrated differences between models across all indices. For the Flesch–Kincaid index, differences were observed among models (*χ* ^2^ = 12.2), with a small‐to‐moderate effect size (Kendall’s *W* = 0.203). Claude 3.7 produced lower (more readable) scores compared with both ChatGPT‐4o (median difference = −2.215, 95% CI: −3.40 to −0.84, *r* = 0.581) and Gemini 2.0 (median difference = −3.485, 95% CI: −4.70 to −2.20, *r* = 0.788), indicating moderate to large effects, while differences between ChatGPT‐4o and Gemini 2.0 were smaller (Table 2, Table S4).

**TABLE 2:** Comparison of the readability levels of the responses of three LLMs (ChatGPT‐4o, Claude 3.7 Sonnet, and Gemini Advanced 2.0 Flash) according to Flesch–Kincaid, Gunning Fog, and Coleman–Liau indices.

Readability	ChatGPT‐4.0	Claude 3.7 sonnet	Gemini advanced 2.0 flash	*χ* ^2^	*p* ^∗^	Effect size (W)
	Mean ± SD	Mean ± SD	Mean ± SD			
	Median [Q1; Q3]	Median [Q1; Q3]	Median [Q1; Q3]			
Flesch–Kincaid	16.3 ± 2.30	14.1 ± 2.68^a^	17.4 ± 2.65^b^	12.2	0.002^∗∗^	0.203
	16.2 [14.7; 17.5]	13.9 [12.3; 16.5]	17.0 [16.1; 19.3]			

Gunning Fog	6.31 ± 1.49	5.00 ± 1.56^a^	7.05 ± 1.21^b^	19.26	< 0.001^∗∗^	0.324
	6.20 [5.13; 7.43]	4.98 [3.69; 6.29]	7.00 [6.26; 7.93]			

Coleman–Liau	13.0 ± 2.43	11.0 ± 2.71^a^	13.8 ± 2.59^b^	11.05	0.003^∗∗^	0.184
	12.2 [11.6; 13.9]	11.4 [9.07; 12.9]	14.0 [11.6; 15.5]			

*Note:* a, b indicate pairwise comparisons.

^a^ChatGPT‐4o vs Claude 3.7.

^b^Claude 3.7 vs Gemini 2.0.

^∗^Friedman test results.

^∗∗^significance at *p* < 0.05. Effect size is reported as Kendall’s W from the Friedman test.

For the Gunning Fog index, model differences were also observed (*χ* ^2^ = 19.47), with a moderate effect size (*W* = 0.324). Claude 3.7 again showed lower scores compared with ChatGPT‐4o (median difference = −1.47, 95% CI: −2.24 to −0.76, *r* = 0.672) and Gemini 2.0 (median difference = −2.235, 95% CI: −2.99 to −1.34, *r* = 0.777), indicating large effects, whereas the difference between ChatGPT‐4o and Gemini 2.0 remained modest (Table 2, Table S4).

Similarly, for the Coleman–Liau index, differences were observed (*χ* ^2^ = 11.05), with a small effect size (*W* = 0.184). Claude 3.7 demonstrated lower readability scores than both ChatGPT‐4o (median difference = −1.87, 95% CI: −3.08 to −0.58, *r* = 0.498) and Gemini 2.0 (median difference = −2.885, 95% CI: −4.03 to −1.56, *r* = 0.691), again indicating moderate to large effects, while differences between ChatGPT‐4o and Gemini 2.0 were less pronounced (Table 2, Table S4).

For reliability and content quality measures, differences between models were less pronounced. For DISCERN scores, variation across models was limited (*χ* ^2^ = 3.33), with a moderate effect size (Kendall’s *W* = 0.417). Although ChatGPT‐4o showed higher median scores compared with Claude 3.7 (median difference = 10.35, 95% CI: 1–17, *r* = 0.668), the confidence intervals overlapped across comparisons, indicating variability and lack of consistent separation between models. According to the DISCERN total scores, ChatGPT‐4o was classified as “excellent,” whereas Claude 3.7 and Gemini 2.0 were classified as “good” (Table 3, Table S5).

**TABLE 3:** Comparison of reliability and content quality scores of three LLMs (ChatGPT‐4o, Claude 3.7 Sonnet, and Gemini Advanced 2.0 Flash) responses assessed by DISCERN and EQIP scales.

Reliability	ChatGPT‐4.0	Claude 3.7 sonnet	Gemini advanced 2.0 flash	*χ* ^2^	*p* ^∗^	Effect size (W)
	Median [Q1; Q3]	Median [Q1; Q3]	Median [Q1; Q3]			
DISCERN	63.5 [62.0; 65.2]	56.5 [47.2; 65.0]^a^	60.5 [58.8; 62.2]	3.33	0.188	0.417
EQIP	58.8 [53.8; 65.0]	55.0 [48.8; 59.6]	55.0 [54.4; 55.6]	0.13	0.935	0.017

*Note:* Effect size is reported as Kendall’s W from the Friedman test.

^a^ChatGPT‐4o vs Claude 3.7.

^∗^Friedman test results.

For EQIP scores, differences between models were minimal (*χ* ^2^ = 0.13), with a negligible effect size (*W* = 0.017). Pairwise comparisons showed small effect sizes and wide confidence intervals, suggesting that all three models performed similarly in terms of content quality. All three LLMs were categorized as “good quality with minor shortcomings” (Table 3, Table S5).

## 4. Discussion

Our findings should be interpreted within the specific context of AI as an educational resource rather than a clinical tool. While we assessed the accuracy and consistency of AI‐generated content against established guidelines, this does not imply readiness for direct clinical application. The educational performance demonstrated here reflects the models’ capacity to convey medical knowledge accurately, which is distinct from the complex requirements of real‐time clinical decision support systems that must account for individual patient variables, dynamic clinical situations, and regulatory considerations.

This is one of the first studies to compare the performance of three leading LLMs in patient education for patients experiencing AMI. While our findings support the utility of LLMs in patient education, they reveal significant performance differences between the models. It is noteworthy that ChatGPT‐4o performs better on the overall assessment, while Claude 3.7 performs better on the treatment process. In technical aspects that require experience and knowledge, such as ECG interpretation, all models performed significantly worse. In the readability evaluation, Claude 3.7 was found to produce more understandable content, which is important for patient education. In terms of content quality and reliability, ChatGPT‐4o was rated “excellent” on the DISCERN scale and “good quality” on the EQIP scale, indicating the superiority of this model in terms of objectivity, comprehensiveness, and reliability of patient information materials.

The significantly higher scores of the ChatGPT‐4o model in terms of content accuracy and reliability compared to other models are in line with the study by King et al., in which the GPT‐4 model achieved 100% accuracy in questions related to heart failure [14]. Similarly, Pay et al. reported that 86% of ChatGPT‐4o’s answers to frequently asked questions about CAD were comprehensive and accurate, compared to 68% for Gemini [15]. Our findings support the potential use of models with GPT‐4.0 architecture for patient education in line with the literature.

In terms of readability, the Claude 3.7 model received significantly lower Flesch–Kincaid, Gunning Fog, and Coleman readability scores than ChatGPT‐4o and Gemini 2.0 in our study. It should be noted that these readability formulas, however, were developed primarily for English texts; therefore, it is not possible to determine precisely at which educational level individuals may find them easy to understand. Sibaa et al. showed that Gemini achieved a higher readability score than ChatGPT 3.5 in patient information content [16]. Karnan et al. showed that ChatGPT used more complex sentence structures than Gemini [17]. In the current literature, no study was found in which Claude 3.7 was directly compared with other models in terms of readability. In our study, Claude 3.7 used a simpler language, whereas ChatGPT‐4o and Gemini 2.0 produced more complex responses.

In terms of DISCERN scores, ChatGPT‐4o obtained the highest score in our study. No statistically significant difference was found between it and Claude 3.7 and Gemini 2.0. This result contradicts the study by Sibaa et al.; in their study, the ChatGPT‐3.5 model showed lower performance in terms of DISCERN score [16]. This difference may be explained by the model versions used (4.0 instead of ChatGPT‐3.5) and the content of the scenarios tested. On the other hand, Karnan et al. reported that ChatGPT and Gemini scored similarly in terms of reliability [17]. Similarly, in Behers et al.’s study, ChatGPT received the highest score in terms of both publication reliability and quality of information on treatment options [18]. These findings align with the observation that ChatGPT‐4 received the highest DISCERN score in our study. In some studies in the literature, EQIP scores of ChatGPT were assessed as having “serious quality‐related problems” or “good quality with minor deficiencies” [8, 19]. In our study, the responses of the three LLMs were generally in the category of “good quality with minor deficiencies” and the highest EQIP score belonged to the ChatGPT‐4o model.

Although it has been reported in the literature that LLM achieves high accuracy rates in ECG analysis [20, 21], it is noteworthy that the answers to the question “What does the ECG show?” received low scores in all three models in our study. This contradiction stems from the fundamental differences between image‐based AI systems and LLMs. As Shu et al. and Patrascanu et al. have shown, although AI models using ECG data are successful in diagnosing STEMI and detecting myocardial dysfunctions, this success relies heavily on image analysis [22, 23]. Similarly, Hillmann et al. reported that the models missed important details in questions with technical content [24]. Brant‐Zawadzki et al. also showed that although ChatGPT‐4 was successful in detecting standard data points, its performance decreased in technical details [25]. This highlights the risk that AI may provide erroneous or incomplete information depending on the quality of the data on which it is trained, as emphasized by Sandeep et al. [20]. In our study, while the ChatGPT‐4o model showed high educational accuracy in the categories of “General information about the disease” and “Diagnostic process,” it was inadequate in technical issues such as ECG interpretation. This finding suggests that there are still limitations in the simplified translation of technical clinical data into patient language.

Hillmann et al. emphasized that all natural language processing‐based chatbots tend to direct the user to the physician for patient questions that require clinical decisions [24]. Similarly, in our study, the ChatGPT‐4o and Gemini models provided more cautious responses that left the final decision to the physician, especially in questions related to treatment, while the Claude model was observed to provide more direct guidance occasionally. This suggests that Claude may make suggestions that exceed ethical limits in some scenarios and, therefore, should be evaluated carefully in this respect. On the other hand, it was found that in some questions in the Gemini model, concepts were confused and answers that disrupted the integrity of meaning, leading the model to receive the lowest accuracy score. This observation is supported by the risk of “confabulation” noted by Hillmann et al. and the finding by Brant‐Zawadzki et al. that the Gemini Ultra model sometimes presented unrealistic information [24, 25]. The ability of chatbots to generate rapid responses may also lead to a tendency to provide superficial content. Pay et al. also reported that although chatbots mostly gave answers in accordance with the guidelines, they provided vague and incomplete information in some questions [15]. All these findings suggest that the use of LLM systems for patient education without physician supervision can be risky, especially when medical accuracy is critical. However, when properly configured, they can play a complementary role in patient education processes. LLMs cannot replace human expertise, but when used correctly, they can provide effective support in patient education processes.

The landscape of myocardial infarction diagnosis and management is undergoing a conceptual shift beyond the traditional STEMI/NSTEMI framework. The emerging occlusion myocardial infarction (OMI) paradigm emphasizes the identification of acute coronary occlusion based on integrative clinical and ECG assessment, rather than relying solely on classic ST‐segment elevation criteria [26, 27]. This paradigm recognizes that coronary occlusion may present with subtle or atypical ECG patterns that would be classified as NSTEMI under traditional criteria, yet require immediate reperfusion therapy. Recent studies have demonstrated both the utility and limitations of AI in this evolving diagnostic framework, showing promise in pattern recognition while highlighting the need for clinical context integration [26, 27].

This paradigm shift has important implications for AI‐based patient education. As diagnostic frameworks become more nuanced, educational tools must adapt to convey increasingly complex clinical reasoning in accessible language. The educational challenges include explaining why some “non‐STEMI” presentations may still warrant emergency intervention, and helping patients understand the integration of multiple clinical variables beyond simple ECG criteria. LLM‐based tools may have particular educational value in this context, as they can potentially synthesize multiple data points and explain integrative reasoning processes. However, our findings—particularly the poor performance on ECG interpretation—underscore that current LLMs are not yet capable of supporting education around these more sophisticated diagnostic paradigms [28].

Future educational frameworks must adapt to these conceptual shifts in cardiovascular care. Rather than positioning our findings as evidence of immediate clinical applicability, we view them as foundational work that informs the development of next‐generation educational tools that can support both patients and learners in navigating evolving standards of care.

Future research should focus on the evaluation of LLM‐generated patient information by real end‐users such as patients and their family members, particularly regarding the readability, comprehensibility, and practical applicability of the content in diverse linguistic and cultural contexts. Conducting similar studies in English‐speaking populations would provide valuable insights into the global generalizability of our findings. In addition, the results of such user‐based evaluations could be systematically shared with LLM developers to improve the quality, clarity, and safety of AI‐assisted patient education tools.

This study evaluated AI models solely as educational tools for patient information. The findings do not address the suitability of these models for direct clinical use, which would require additional evaluations, including real‐time performance, integration with clinical workflows, patient safety protocols, and regulatory compliance. Additional limitations include that only the AMI scenario was evaluated, and LLM responses were analyzed only once; as GAI outputs are probabilistic in nature, results may vary across sessions, which may limit the reproducibility of the findings. The question set used in this study was not formally validated against the actual priorities of ED patients and their families. Future studies should involve patient and family advisors in the question development process to ensure ecological validity and alignment with real‐world informational needs. In addition, the continuous updating of the models used may lead to different results in the future. Strengths include the comparison of three different models in the same scenario, the use of validated scales, and the design of questions that reflect real patient language. The evaluations conducted by associate professors of emergency medicine enhanced the reliability of the findings. Overall, this study represents the first direct comparison of three state‐of‐the‐art LLMs in an emergency myocardial infarction education context, shedding light on their potential role in patient education and providing direction for future AI‐assisted communication in healthcare.

## 5. Conclusion

In our study, we evaluated the performance of three different LLMs responding to patient questions about AMI as educational tools. ChatGPT‐4o received the highest scores in terms of content accuracy and reliability; Claude 3.7 produced more comprehensible answers, while Gemini 2.0 experienced concept confusion in some questions. While the models were successful in general knowledge and the diagnostic process, they were inadequate in technical questions like ECG interpretation. This suggests that LLM systems still have limitations in translating clinical knowledge into patient language. Our findings suggest that LLMs show promise in patient education, but should be supervised by a physician. These findings are exploratory and hypothesis‐generating, informing future curriculum design and evaluation frameworks rather than immediate implementation. Especially in critical settings such as EDs, LLM systems should be seen as a supportive educational tool, and final educational guidance and clinical decisions should always be guided by healthcare professionals.

## Author Contributions

The author solely conceptualized and designed the study and conducted the material preparation, data collection, and analysis. The first draft of the manuscript was written by Korkmaz İ.

## Funding

No financial support was received for this study.

The author did not receive support from any organization for the submitted work.

## Disclosure

A preprint version of this manuscript has been previously published on Research Square and is available at: https://www.researchsquare.com/article/rs-6433953/v1 [26]. The author reviewed and approved the final version of the manuscript.

## Ethics Statement

This study was approved by the Izmir City Hospital Institutional Review Board/Ethics Committee (Date/Approval No: 2026/144). Informed consent was obtained from all evaluators who participated in the assessment of AI‐generated responses.

## Consent

This study did not involve any individual person’s data in any form (including individual details, images, or videos), and no consent for publication was required.

## Conflicts of Interest

The author declares no conflicts of interest.

## Supporting Information

Additional supporting information can be found online in the Supporting Information section.

## Supporting information


**Supporting Information** The supporting file includes the complete list of patient questions posed to the large language models (LLMs) and the scoring given by the evaluators for each response.

## Data Availability

The datasets generated and/or analyzed during the current study are not publicly available in a repository but are available from the corresponding author upon reasonable request.

## Appendix A

The dataset prepared in R and the analysis scripts can be shared by the author upon reasonable request.
