# Supplementary material for: Accuracy and Reliability of AI Models in Emergency Myocardial Infarction Education
Source: Emerg Med Int. 2026 Jun 13;2026:5530861. doi: 10.1155/emmi/5530861 (PMC13263638; doi:10.1155/emmi/5530861)
Supplement: Supplementary file 1 — Supporting Information The supporting file includes the complete list of patient questions posed to the large language models (LLMs) and the scoring given by the evaluators for each response. [file EMMI-2026-5530861-s001.docx]

Supplementary Material

# 1. Prompt Template and Methodology

All large language models (ChatGPT-4o, Claude 3.7 Sonnet, and Gemini Advanced 2.0 Flash) were queried using the following standardized prompt template. Each model was provided with the identical clinical scenario and ECG image, followed by individual questions from the question set.

## 1.1 Initial Scenario Prompt

**Clinical Scenario Presented to All Models:**

"Hello, I am the son of a 56-year-old male patient. My father has a history of diabetes and hypertension. He was brought to the emergency room about two hours ago with severe chest pressure, pain radiating to his arm and jaw, nausea, and vomiting. The doctors have performed some blood tests and taken an electrocardiogram, and they mentioned that a complete blood count, biochemistry, troponin, and CK-MB tests are being done. He was given 300 mg of aspirin and 5000 units of heparin intravenously. We were also informed that an emergency coronary angiography is planned. I am very worried and have some questions. Could you please explain to me, at the level of an expert doctor, what is happening and what we should expect?"

## 1.2 ECG Image

Along with the scenario, an inferior STEMI ECG image from Life in the Fastlane website was uploaded to each model interface (although visual processing capabilities vary by model)


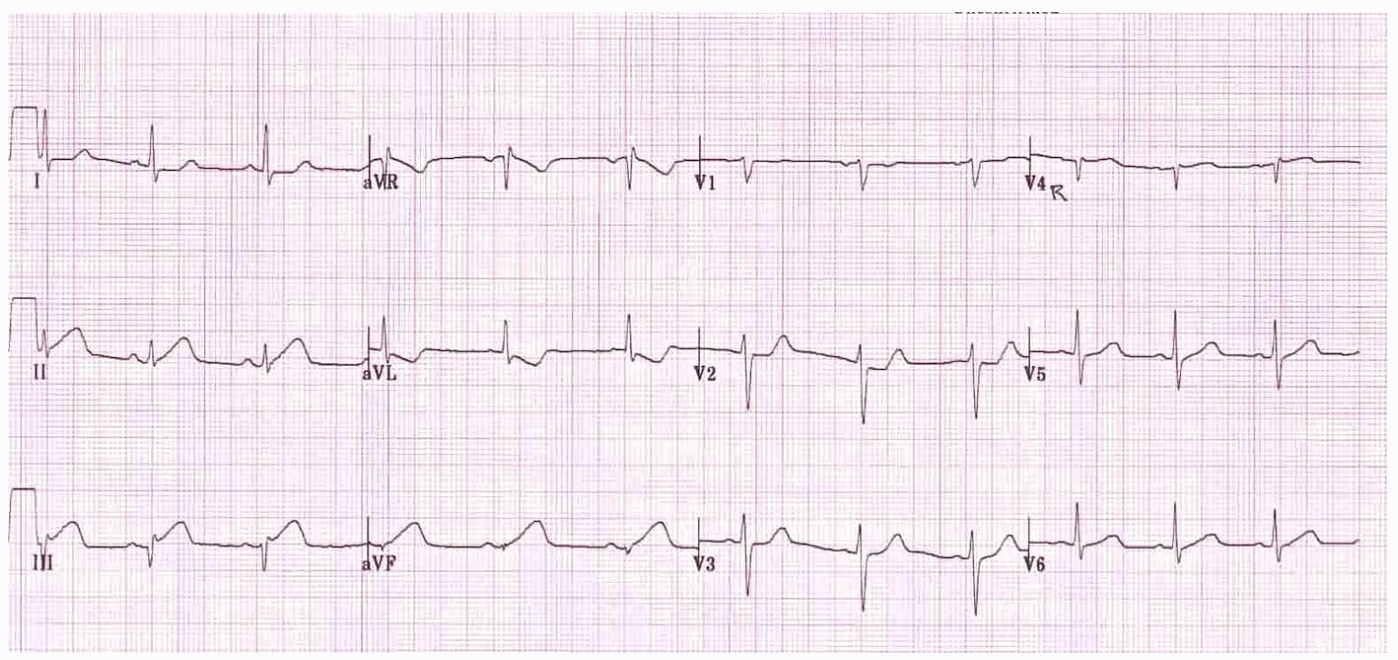


**References:** Burns E, Buttner R, Buttner EB and R. Inferior STEMI [Internet]. Life in the Fast Lane • LITFL. 2018 [cited 2025 Apr 3]. Available from: https://litfl.com/inferior-stemi-ecg-library/

## 1.3 Question Administration

Following the initial scenario, each of the 30 questions (organized under three domains: general disease information, diagnostic process, and treatment process) was presented individually to each model.

**Methodological Details:**

- All LLM sessions were initiated fresh for each question to prevent conversation history from influencing subsequent responses
- Default model parameters were used for all three LLMs without modification
- No word or character limits were applied to allow unrestricted comprehensive answers
- All questions were posed in Turkish as presented in Table S1

# 2. Example Question-Response Sets

Below are three representative examples showing how each model responded to selected questions from different domains. These examples illustrate the variation in response style, length, and technical detail across models. All responses are presented in Turkish as originally generated.

## 2.1 Example from General Disease Information Domain

This question received high scores across all models, demonstrating strong performance in explaining clinical concepts in accessible language.

**Question:** "Neden göğüs ağrısı dışında kola ve çeneye de ağrı yayılıyor?" ("Why does the pain spread to the arm and jaw in addition to the chest?")

*ChatGPT-4o Response (Score: 4.75/5):*

Kalp krizinde göğüs ağrısının kola ve çeneye yayılmasının nedeni, kalbi besleyen damarların (koroner arterler) tıkanması sonucu kalp kasının yeterince oksijen alamamasıdır. Bu durum sinir uçlarını uyararak ağrının farklı bölgelere yayılmasına neden olabilir. Kalp ve bu bölgelerdeki sinirler arasındaki bağlantılar nedeniyle ağrı, göğüs bölgesinin yanı sıra sol kol, çene ve hatta sırta da yayılabilir.

*Claude 3.7 Sonnet Response (Score: 4.75/5):*

Bu ağrıların yayılmasının nedeni, kalbinizden gelen ağrı sinyallerinin sinir sisteminizde farklı bölgelere ulaşmasıdır. Buna "yansıyan ağrı" denir. Kalp, göğüs, sol kol ve çene bölgesinin sinir ağları birbiriyle bağlantılıdır. Bu yüzden kalpteki bir sorun: • Sol kola • Çeneye • Boyun bölgesine • Sırta • Bazen sağ kola bile yayılabilir. Beyin bu sinyalleri tam olarak lokalize edemediği için ağrıyı bu bölgelerde de hissedersiniz. Bu yayılan ağrı paterni kalp krizi için tipiktir ve tanı koymada önemli bir bulgudur.

*Gemini Advanced 2.0 Flash Response (Score: 4.00/5):*

Kalp krizi sırasında göğüs ağrısının kola ve çeneye yayılmasının nedeni, kalbin ve bu bölgelerin sinirlerinin aynı sinir ağı üzerinden beyne sinyal göndermesidir (viseral-somatik ağrı yayılımı). Kalp, göğüs, kol ve çene bölgeleri ortak sinir yollarını (örneğin, C5-T4 segmentlerini) paylaşır. Beyin, bu sinyalleri yanlış yorumlayarak ağrıyı farklı bölgelerde hissediyormuşsunuz gibi algılar. Bu, kalp krizi için tipik bir bulgudur.

## 2.2 Example from Diagnostic Process Domain

This question received the lowest scores across all models, highlighting current limitations in technical ECG interpretation and image analysis capabilities.

**Question:** "EKG'de ne görünüyor? Normal mi?" ("What does the ECG show? Is it normal?")

*ChatGPT-4o Response (Score: 3.25/5):*

EKG'nizde görülenler normal değil. Bu EKG, bir kalp krizi (miyokard enfarktüsü) sırasında görülen tipik değişiklikleri gösteriyor. EKG'deki Anormallikler: • ST segment elevasyonu: EKG'nin bazı bölgelerinde ST segmentinde yükselme görülüyor. Bu, kalp kasında hasar olduğunu gösteren önemli bir işarettir. Özellikle V2 ve V3 derivasyonlarında belirgin ST elevasyonu mevcut. • Q dalgaları: Bazı derivasyonlarda (örneğin V2, V3) Q dalgaları görülüyor. Q dalgaları, eski bir kalp krizinin veya mevcut bir kalp krizinin işareti olabilir. • T dalgası değişiklikleri: T dalgalarında da anormallikler görülüyor. Bu da kalp kasının durumundan kaynaklanıyor. Bu bulgular, akut bir kalp krizini düşündürüyor.

*Claude 3.7 Sonnet Response (Score: 1.75/5):*

EKG'niz kesinlikle normal değil. Akut kalp krizi bulgularını net olarak gösteriyor. Teknik olarak açıklayayım: V1-V6 derivasyonlarında (göğüs derivasyonları) belirgin ST segment yükselmesi var. Bu bulgu: • Kalbin ön duvarında (anterior) bir kalp krizi olduğunu • Sol ön inen koroner arterde (LAD) tıkanıklık olduğunu gösteriyor.

*Gemini Advanced 2.0 Flash Response (Score: 2.50/5):*

Şu an manuel olarak ST segment yükselmesi, T dalga inversiyonu veya iskemik değişiklikleri değerlendirebilirim. Ön değerlendirmeye göre ST segmentinde yükselmeler mevcut olabilir, ancak kesin yorum için doktorunuzun klinik değerlendirmesi ve kan testlerinizle birlikte incelenmesi gerekir. Eğer ST segment yükselmesi varsa, bu STEMI (ST Elevation Myocardial Infarction) yani büyük bir kalp krizi olabilir ve acil anjiyografi gereklidir.

## 2.3 Example from Treatment Process Domain

This question demonstrates how models handle treatment-related inquiries with varying levels of directness and clinical detail.

**Question:** "Anjiografide stent mi takılacak?" ("Will a stent be placed during angiography?")

*ChatGPT-4o Response (Score: 4.50/5):*

Anjiyografi sonucunda stent takılıp takılmayacağı, damarlarınızdaki darlıkların veya tıkanıklıkların durumuna göre belirlenir. Anjiyografi sırasında doktorunuz damarlarınızı detaylı inceleyecek ve aşağıdaki durumlarda stent takılmasına karar verebilir: • Damarlarda darlıklar: Damar çapının %70'inden fazlasını tıkayan darlıklar genellikle stent takılmasını gerektirir. • Kalp krizine neden olan tıkanıklıklar: Kalp krizine yol açan akut tıkanıklıkların açılması için acil stent takılması gerekebilir. • İlaç tedavisine yanıt vermeyen darlıklar: İlaç tedavisine rağmen devam eden göğüs ağrısı veya diğer belirtiler varsa, stent takılması düşünülebilir.

*Claude 3.7 Sonnet Response (Score: 4.25/5):*

Anjiyografi sırasında damarınızın durumuna göre karar verilecek: 1. Önce damarlar görüntülenecek: • Tıkanıklığın yeri • Tıkanıklığın derecesi • Damarın yapısı değerlendirilecek. 2. Sonra işlem yapılacak: • Genellikle balon ile damar genişletilir • Çoğu durumda stent takılır • Bazen sadece balon yeterli olabilir. 3. Stent: • Metal küçük bir kafes • Damarı açık tutar • Yeniden tıkanmayı önler • Günümüzde çok güvenli. 4. İşlem sonrası: • Kan sulandırıcı ilaçlar başlanır • Stentin içinde pıhtı oluşması önlenir • Düzenli kontroller yapılır. Kesin kararı anjiyografi sırasında doktorunuz verecek.

*Gemini Advanced 2.0 Flash Response (Score: 4.00/5):*

Anjiyografi sırasında damarlarınıza kontrast madde verilerek tıkanıklığın yeri ve şiddeti tespit edilecek. Stent takılıp takılmayacağı, anjiyografi sonucuna göre karar verilecek. 1. Anjiyografi Sonucuna Göre 3 Olası Durum: 1. Damar Hafif Daralmışsa (Tıkanıklık %50'nin Altında) • Stent gerekmez. • İlaç tedavisi ve yaşam tarzı değişiklikleriyle takip edilir. 2. Damar Ciddi Derecede Daralmışsa (%50-90 Arasında) • Balon anjiyoplasti ile damar genişletilir ve stent yerleştirilir. • Stent, damarı açık tutarak yeniden tıkanmayı önler. 3. Damar Tamamen Tıkalıysa (%100 Tıkanıklık – Akut Kalp Krizi Durumu) • Acil olarak balon ve stent takılması gerekir.

# 3. Complete Question List and Accuracy Scores

**Table S1.** Accuracy scores for all 30 patient education questions by domain and large language model

The table below presents all 30 questions organized by domain, along with the accuracy scores (5-point Likert scale) assigned by the expert panel for each model's response. Questions are presented in English translation with Turkish originals available upon request.

|  | GEMİNİ 2.0 | CHATGPT-4.0 | CLAUDE 3.7 |
| --- | --- | --- | --- |
| GENERAL INFORMATION ABOUT THE DISEASE |  |  |  |
| What exactly is happening to my father? Is it really a heart attack? | 3,75 | 4,25 | 4 |
| Why is he having a heart attack? He seemed fine before. | 3,5 | 4 | 4,25 |
| Is it normal to have a heart attack at his age? | 4 | 4,5 | 4,25 |
| Why does the pain spread to his arm and jaw in addition to the chest? | 4 | 4,75 | 4,75 |
| He also has nausea and vomiting—is that related to the heart attack or something else? | 4 | 4,5 | 3,5 |
| Is his condition serious? Is his life in danger? | 4 | 4,25 | 3,5 |
| Are you sure it’s a heart attack? Could it be something else? | 4 | 4,25 | 4,25 |
| How urgent is his situation? Is it possible to wait a little before making decisions? | 4,25 | 5 | 5 |
| Should we consider transferring him to another hospital or consult another doctor? | 3,5 | 4,5 | 4,25 |
| No one in our family has experienced anything like this before. Why is this happening to him? | 4 | 4,25 | 4,5 |
| DIAGNOSTIC PROCESS |  |  |  |
| What does his ECG show? Is it really abnormal? | 2,5 | 3,25 | 1,75 |
| What are you checking for in his blood tests? | 3,5 | 3,5 | 3,75 |
| Why is angiography necessary? Is it a risky procedure? | 3,5 | 4,25 | 4,25 |
| How much damage has already happened to his heart? Can this be determined now? | 4,25 | 4,5 | 4,25 |
| Will the tests he’s undergoing be painful? How long will they take? | 4,5 | 4,5 | 4,5 |
| Is it necessary to perform the angiogram immediately? | 4,25 | 5 | 4,25 |
| Is there any chance of a misdiagnosis? | 3,75 | 4,5 | 4 |
| What happens if treatment isn’t started right away? | 3,5 | 4,25 | 4,5 |
| Could imaging methods like CT or MRI be used instead of angiography? | 4 | 4,75 | 4 |
| Can such a critical decision really be made so quickly? | 4,25 | 4,75 | 4,5 |
| TREATMENT PROCESS |  |  |  |
| What medications has he been given so far? | 4,5 | 4,5 | 4,25 |
| Will a stent be placed during the angiography? | 4 | 4,5 | 4,25 |
| Is there a chance he’ll need bypass surgery? | 4,25 | 4 | 4 |
| What are the risks of these blood thinners you’re using? | 4 | 4,5 | 4 |
| Are there any side effects we should expect during treatment? | 4,25 | 4,5 | 3,75 |
| Will his heart recover fully after all this? | 4 | 4,75 | 4,25 |
| Isn’t there any alternative treatment other than angiography? | 3,75 | 4 | 4 |
| Is it possible that medication alone could be enough? | 3,5 | 4 | 3,75 |
| Is there a risk that he might die during the procedure? | 3,75 | 4,5 | 4,25 |
| When will he be able to return to his normal life? | 4,5 | 4,5 | 4,25 |

# 4. Gamer Checklist

**Table S2.**Gamer Checklist (1)

| No | Item | Reported | Page | |
| --- | --- | --- | --- | --- |
| 1 | Did you use any GAI tools (such as large language models or large visual models) in any section or step of this manuscript or study? | YES | Generative AI tools (ChatGPT-4o, Claude 3.7 Sonnet, and Gemini Advanced 2.0 Flash) were used as the primary subject of this study; their responses to clinical patient education questions were systematically evaluated. These tools were not used in any phase of manuscript preparation, language editing, data analysis, or study design | |
| 2 | Specify the GAI tool(s) used, their versions and/ or release dates and the date(s)/period the tools were used. | YES | Three GAI tools were used in this study, all accessed via their official web interfaces on February 25, 2025:  1. ChatGPT-4o (OpenAI; chat.openai.com; accessed February 25, 2025) 2. Claude 3.7 Sonnet (Anthropic; claude.ai; accessed February 25, 2025) 3. Gemini Advanced 2.0 Flash (Google; gemini.google.com; accessed February 25, 2025)  All tools were accessed through their standard front-end web interfaces. Default model parameters were applied without modification, including temperature settings. Exact session timestamps were not recorded; however, all sessions were conducted within the same calendar day (February 25, 2025). | |
| 3 | Describe whether a specific prompting technique was used to generate any content of the manuscript or to perform analyses during the study. Please also provide the unedited responses to the prompts. | YES | A standardised two-step prompting approach was applied. First, the clinical scenario and ECG image were uploaded to each model (Section 1.1–1.2). Subsequently, 30 questions were posed individually in fresh sessions (Section 1.3). No advanced prompt engineering techniques were used. Representative unedited model responses are provided in Section 2 of this Supplement. Full response datasets are available from the corresponding author upon reasonable request. | |
| 4 | If a new GAI tool was developed or fine-tuned based on an existing AI model, report the name and version of the original model. | N/A | No new GAI tool was developed or fine-tuned in this study. Existing commercial models (ChatGPT-4o, Claude 3.7 Sonnet, and Gemini Advanced 2.0 Flash) were used as provided, without any modification to their underlying architecture or training. | |
| 5 | Describe the role of GAI tools in all phases of this study where they were used (including manuscript writing). | YES | GAI tools served exclusively as the subject of evaluation in this study. Their role was limited to generating responses to 30 patient education questions about acute myocardial infarction within a standardised clinical scenario. The tools were not used in study design, data collection, statistical analysis, manuscript writing, or language editing. All phases of the research process were conducted independently by the author. | |
| 6 | Report the specific section or paragraphs of the manuscript that GAI tools contributed to. | N/A | GAI tools did not contribute to any section or paragraph of this manuscript. The tools were used solely as research subjects. All manuscript sections, including introduction, methods, results, discussion, and conclusion, were written entirely by the author without GAI assistance. | |
| 7 | Describe how the content generated by GAI tools was verified and (when necessary) modified. | YES | All GAI-generated responses were systematically verified by a panel of four emergency medicine associate professors with 10–20 years of clinical experience. Each response was independently evaluated against current acute coronary syndrome guidelines (2023 ESC Guidelines and 2025 ACC/AHA Guidelines) using a validated 5-point Likert scale. In cases of scoring disagreement, evaluators held group discussions to reach consensus. Responses were not modified; instead, inaccuracies were recorded as part of the accuracy scoring process. Spelling and grammatical errors were reviewed prior to evaluation, and no significant errors affecting meaning were detected. | |
| 8 | Describe how data privacy and confidentiality were ensured during the use of GAI tools. | YES | No real patient data, personally identifiable information, or sensitive clinical records were entered into any GAI tool interface. The clinical scenario used in this study was entirely fictional and constructed by the author solely for research purposes. The ECG image used was sourced from a publicly available educational website (Life in the Fast Lane, LITFL) and contains no patient-identifiable information. All interactions with GAI tools involved only this anonymised fictional scenario and standardised questions, ensuring full compliance with data privacy requirements. | |
| 9 | Describe whether and how the use of GAI tools may have influenced the interpretation of results, the study’s overall accuracy, or conclusions. | YES | Since GAI tools were the primary subject of evaluation rather than analytical instruments, their use inherently shaped the results and conclusions of this study. The accuracy, reliability, and readability of GAI-generated responses directly determined the study outcomes. To minimise interpretation bias, responses were evaluated by four blinded emergency medicine associate professors using validated, standardised instruments (5-point Likert scale, DISCERN, EQIP). Evaluators were blinded to model identity throughout the assessment process. However, it should be noted that the models' outputs are probabilistic and may vary across sessions; as responses were collected in a single session, results may not be fully reproducible. This limitation has been acknowledged in the Discussion section. | |
| AI, artificial intelligence; GAI, generative artificial intelligence; GAMER, Generative Artificial intelligence tools in MEdical Research; N/A, not applicable. | | | |  |

1. Luo X, Tham YC, Giuffrè M, Ranisch R, Daher M, Lam K, et al. Reporting guideline for the use of Generative Artificial intelligence tools in MEdical Research: the GAMER Statement. BMJ Evidence-Based Medicine. 2025 Dec 1;30(6):390–400. doi:10.1136/bmjebm-2025-113825 PubMed PMID: 40360239.

**Table S3.** Pairwise comparisons of LLM performance across domains

| Domain | Comparison | Median Difference | 95% CI | Effect Size (r) |
| --- | --- | --- | --- | --- |
| Total Score | ChatGPT-4o vs Claude 3.7 | 0.375 | 0.25–0.50 | 0.614 |
|  | ChatGPT-4o vs Gemini 2.0 | 0.50 | 0.50–0.625 | 0.803 |
|  | Claude 3.7 vs Gemini 2.0 | 0.25 | 0–0.50 | 0.358 |
| General Information | ChatGPT-4o vs Claude 3.7 | 0.25 | 0–0.625 | 0.416 |
|  | ChatGPT-4o vs Gemini 2.0 | 0.50 | 0.375–0.75 | 0.879 |
|  | Claude 3.7 vs Gemini 2.0 | 0.375 | −0.125–0.75 | 0.554 |
| Diagnostic Process | ChatGPT-4o vs Claude 3.7 | 0.39 | 0–1.00 | 0.561 |
|  | ChatGPT-4o vs Gemini 2.0 | 0.75 | 0.625–0.75 | 0.810 |
|  | Claude 3.7 vs Gemini 2.0 | 0.25 | −0.25–0.75 | 0.370 |
| Treatment Process | ChatGPT-4o vs Claude 3.7 | 0.375 | 0.25–0.50 | 0.796 |
|  | ChatGPT-4o vs Gemini 2.0 | 0.50 | 0.125–0.625 | 0.694 |
|  | Claude 3.7 vs Gemini 2.0 | 0.00 | −0.25–0.25 | 0.059 |
| Values represent median differences with 95% confidence intervals (CI). Effect sizes are reported as rank-biserial correlation coefficients (r). | | | | |

**Table S4.** Pairwise comparisons of readability indices

| Index | Comparison | Median Difference | 95% CI | Effect Size (r) |
| --- | --- | --- | --- | --- |
| Flesch–Kincaid | ChatGPT vs Claude | 2.215 | 0.84–3.40 | 0.581 |
|  | ChatGPT vs Gemini | −1.12 | −2.46–0.11 | 0.319 |
|  | Claude vs Gemini | −3.485 | −4.70–−2.20 | 0.788 |
| Gunning Fog | ChatGPT vs Claude | 1.47 | 0.76–2.24 | 0.672 |
|  | ChatGPT vs Gemini | −0.755 | −1.45–0.27 | 0.304 |
|  | Claude vs Gemini | −2.235 | −2.99–−1.34 | 0.777 |
| Coleman–Liau | ChatGPT vs Claude | 1.87 | 0.58–3.08 | 0.498 |
|  | ChatGPT vs Gemini | −0.875 | −2.23–0.59 | 0.227 |
|  | Claude vs Gemini | −2.885 | −4.03–−1.56 | 0.691 |

**Table S5.** Pairwise comparisons of DISCERN and EQIP scores

| Scale | Comparison | Median Difference | 95% CI | Effect Size (r) |
| --- | --- | --- | --- | --- |
| DISCERN | ChatGPT vs Claude | 10.35 | 1–17 | 0.668 |
|  | ChatGPT vs Gemini | 3.285 | −2–9 | 0.552 |
|  | Claude vs Gemini | −4.75 | −13–8 | 0.444 |
| EQIP | ChatGPT vs Claude | 2.366 | −2.5–11.25 | 0.276 |
|  | ChatGPT vs Gemini | 6.608 | −5–20 | 0.272 |
|  | Claude vs Gemini | −0.782 | −20–13.3 | 0.000 |
